# Supplementary material for: Structural insights into the in situ assembly of clustered protocadherin γB4
Source: Nat Commun. 2025 Feb 16;16:1682. doi: 10.1038/s41467-025-56948-x (PMC11830823; doi:10.1038/s41467-025-56948-x)
Supplement: Supplementary file 1 — Supplementary Information [file 41467_2025_56948_MOESM1_ESM.pdf]

Supplementary figures

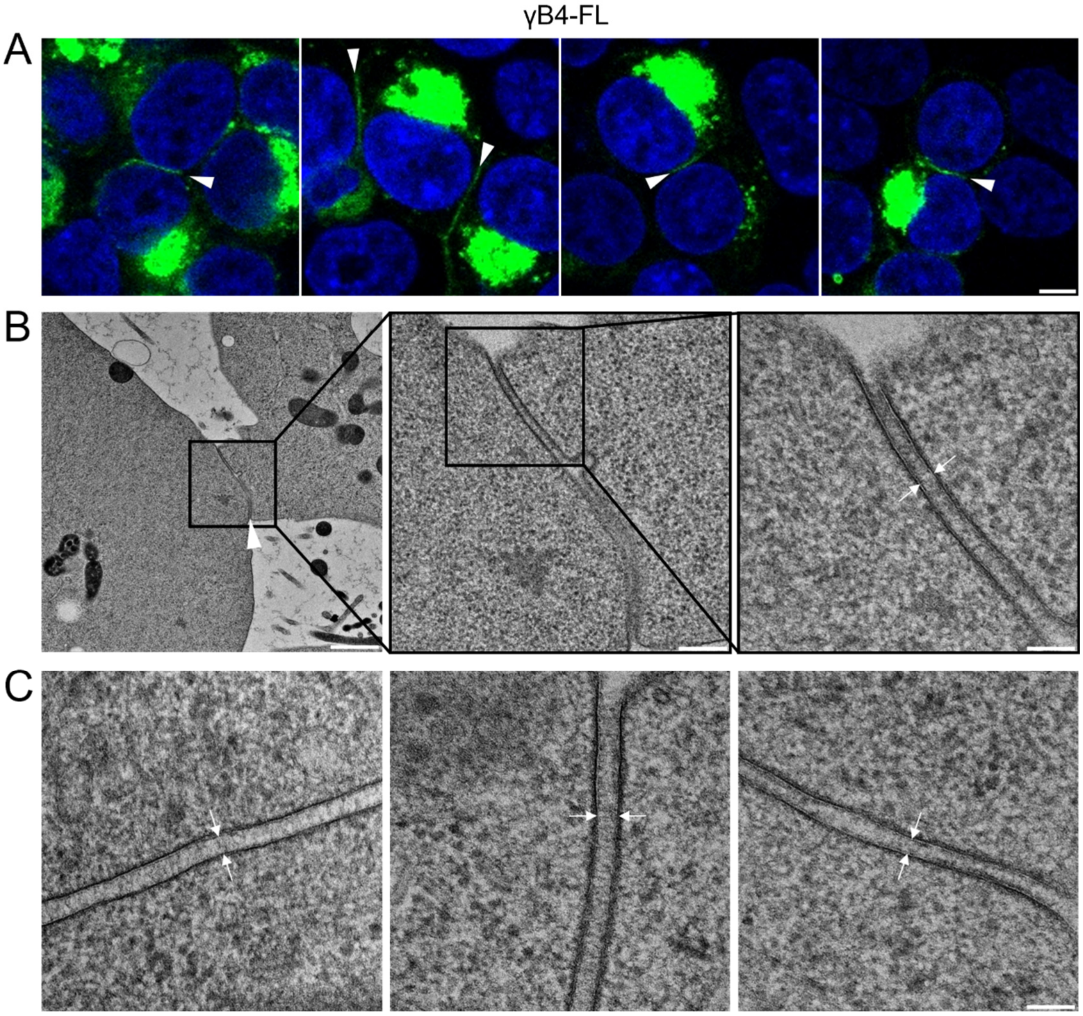

Supplementary Figure 1. Microscopic images of the cell adhesion interfaces by γB4-FL

(A) Confocal fluorescent images of adhesion interfaces (white arrowheads) by γB4-FL Scale bar, 5 μm.

(B) EM images of an adhesion interface (white arrowhead, left) by γB4-FL and the zoom-in views (middle and right). Scale bar, 1 μm (left); 250 nm (middle); 100 nm (right).

(C) A gallery of the γB4-FL mediated adhesion interfaces (white arrows). Scale bar, 100 nm.

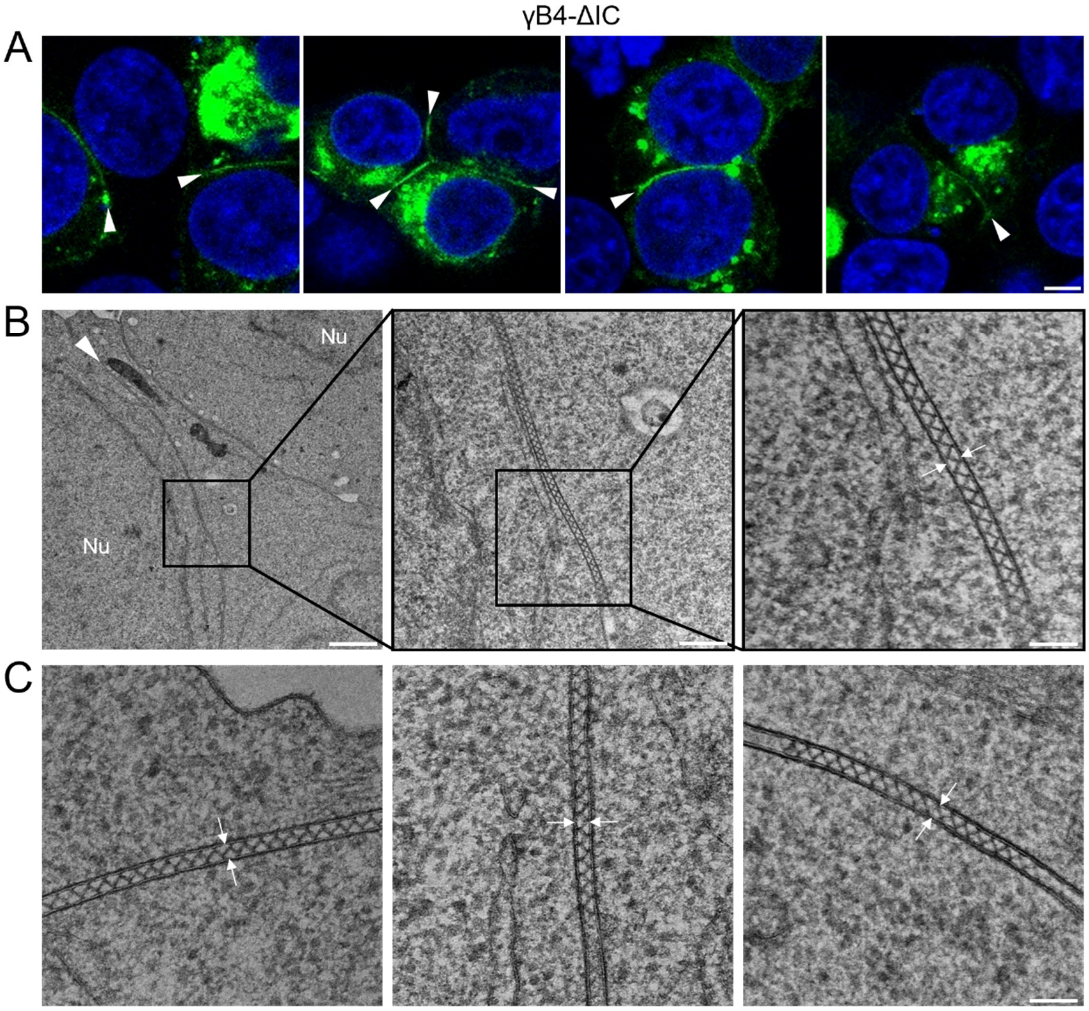

Supplementary Figure 2. Microscopic images of the cell adhesion interfaces by γB4-ΔIC

(A) Confocal fluorescent images of adhesion interfaces (white arrowheads) by γB4-ΔIC. Scale bar, 5 μm.

(B) EM images of an adhesion interface (white arrowhead, left) by γB4-ΔIC and the zoom-in views (middle and right). Scale bar, 1 μm (left); 250 nm (middle); 100 nm (right).

(C) A gallery of the γB4-ΔIC mediated adhesion interfaces (white arrows). Scale bar, 100 nm.

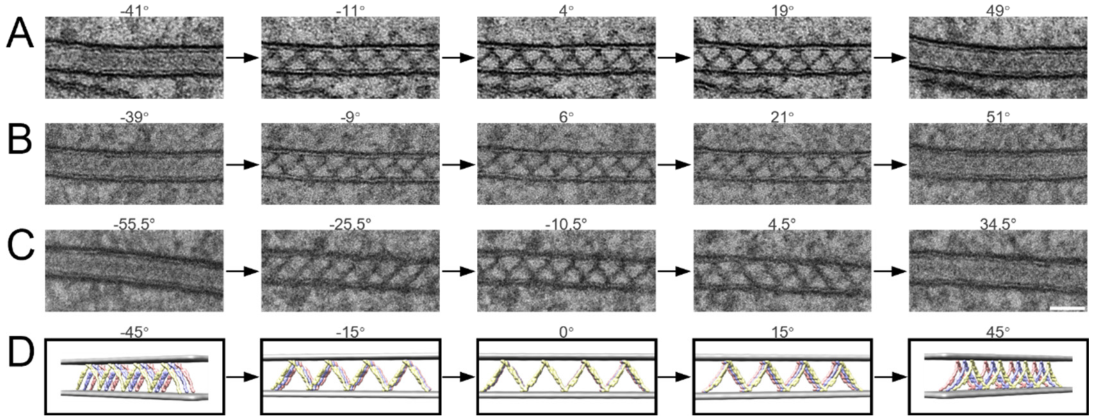

Supplementary Figure 3. Tomographic tilt series of the cell adhesion interfaces by γB4-ΔIC

(A-C) Three tomographic tilt series of the cell adhesion interfaces by γB4-ΔIC visualized at different tilt angles. Scale bar, 50 nm.

(D) The 3D assembly model of γB4-ΔIC visualized at the corresponding tilt angles shown in (A-C).

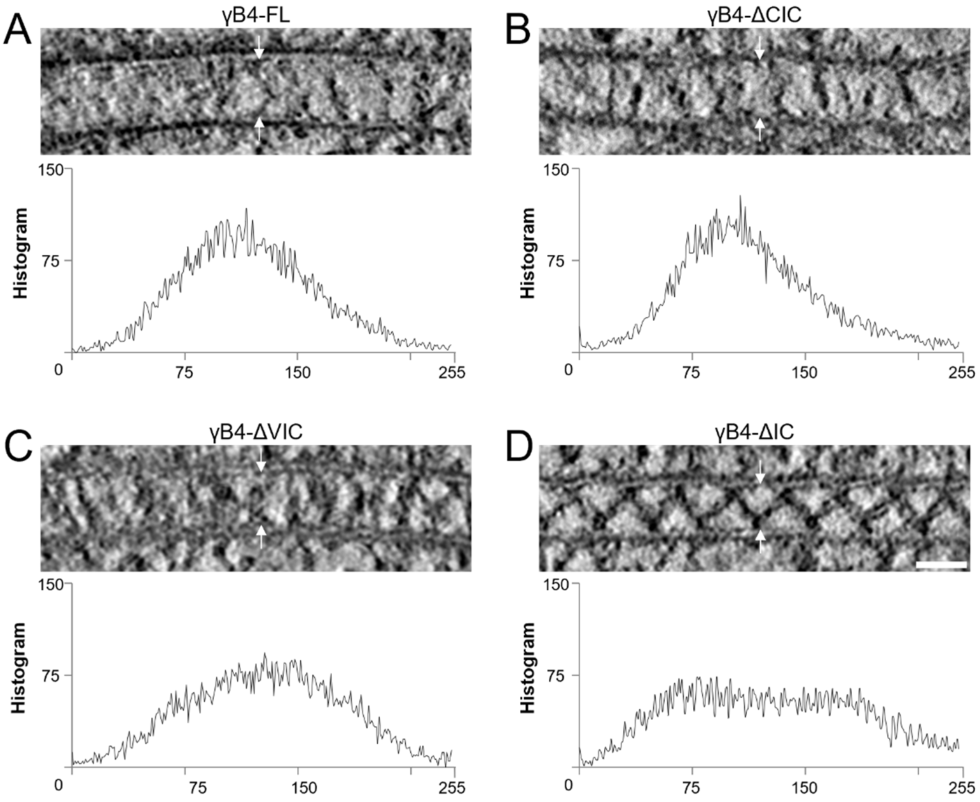

Supplementary Figure 4. Histograms of the intermembrane tomographic densities of the interfaces by γB4-FL and the IC-truncation mutants of γB4

(A) A tomographic slice of the γB4-FL mediated interface (top, also shown in Fig. 1C) and the corresponding histogram (bottom).

(B) A tomographic slice of the γB4-ΔCIC mediated interface (top, also shown in Fig. 7B) and the corresponding histogram (bottom).

(C) A tomographic slice of the γB4-ΔVIC mediated interface (top, also shown in Fig. 7D) and the corresponding histogram (bottom).

(D) A tomographic slice of the γB4-ΔIC mediated interface (top, also shown in Fig. 2C) and the corresponding histogram (bottom).

Scale bar, 35 nm.

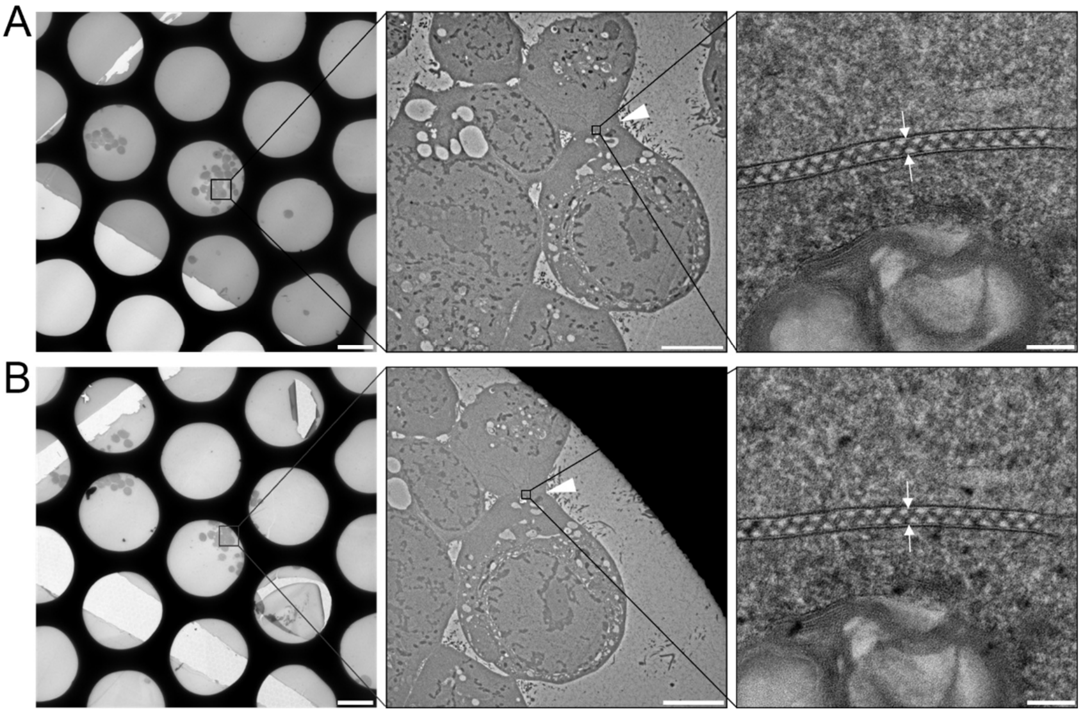

Supplementary Figure 5. EM sections of a γB4-ΔIC mediated interface

(A-B) Two continuous sections of a γB4-ΔIC mediated interface (white arrows). Scale bar, 50 μm (left); 5 μm (middle); 100 nm (right).

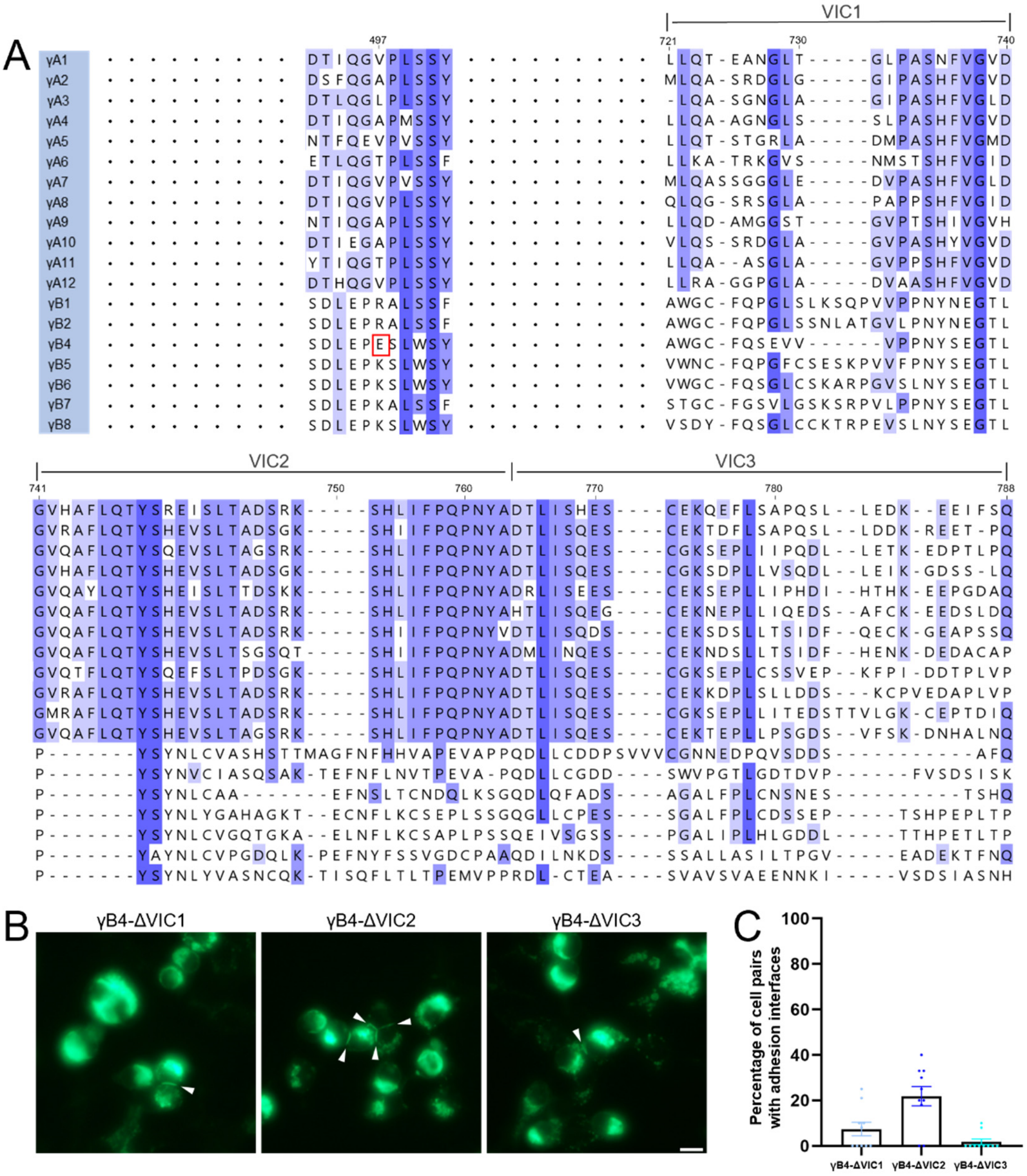

Supplementary Figure 6. Sequence alignment and mutants of the VIC region of γB4

(A) Sequence alignment of γ subfamily members of cPcdh around amino acid position 497 and VIC region.

(B) The fluorescent images of cell adhesion mediated by γB4-ΔVIC1, γB4-ΔVIC2 and γB4-ΔVIC3. The adhesion interfaces are indicated by white arrowheads (scale bar, 15 μm).

(C) The statistics of the adhesion interfaces by γB4-ΔVIC1, γB4-ΔVIC2 and γB4-ΔVIC3. Each dot represents the percentage of highlighted fluorescent interfaces appeared in the pairs of neighboring cells in a stochastic field of view. A total of

ten views were collected for each construct. The data are plotted as mean ± SE and also provided as a source data file.

Supplementary Table 1. Primers for the constructs (5'-3' direction)

Constructs	Forward primer	Reverse primer
γB4-FL	gatcggaaaacctctcgagccaccatgcgggagggc	catagatccactacccttcttctctttcttgcccga
γB4-ΔIC	gatcggaaaacctctcgagccaccatgcgggagggc	catagatccactacccttcctgctggaggagtg
GFP	ggtagtggatctatggtgagcaagggc	gtcgaggtcgggggatccttacttgtacagctcgtc
mCherry	aagaagggtagtggatct	gtcgaggtcgggggatccttacttgtacagctcgtc
EC5 of γB6	gtgaacgataacgctccggctttc	ggcgcgttgtcgttgcggtcacc
EC6 of γB6	caacgacaacgcgccacgcgtg	gcaggacctcctgcaagctatcag
EC5-6 of γB6	gtgaacgataacgctccggctttc	gcaggacctcctgcaagctatcag
T451Q/V453S	aacgccccagttttccagaagagttcatacctggtccatgt	acatggaccaggtatgaactcttctggaaaactggggcgtt
Q484H/Y488S	gatttgggggccaatggccatgttacctattccatcatagc	gctatgatggaataggtaacatggccattggcccccaaatc
E497K	agcgacctggagcctaaatctctgtggtcctacg	cgtaggaccacagagatttaggctccaggtcgct
H535Q/S537K	caggcgcgggaccaaggaaagcccacactcagcg	cgctgagtgtgggctttccttggtcccgcgcctg
L585A	ccgagcccggatacgcggtcaccaaggtgg	ccaccttggtgaccgcgtatccgggctcgg
V590G	ggtcaccaaggtgggggctgtggatgcag	ctgcatccacagcccccaccttggtgacc
TM-IC	ttctgccccacgctctctgctgacagcctgcaagag	ctcttgcaggctgtcagcagagagcgtggggcagaa
TM-VIC	tgagtcaacctcccatcagggtagtggatctatggtgag	ctcaccatagatccactaccctgatgggaggttgactca
TM-CIC	cactcctccagcaggaagcaagccccgcccaac	gttgggcggggcttgcttcctgctggaggagtg
IC	acctctcgagccaccgcatggggctgcttt	aaagcagccccatgcggtggctcgagaggt
γB4-ΔCIC	tgagtcaacctcccatcagggtagtggatctatggtgag	ctcaccatagatccactaccctgatgggaggttgactca
γB4-ΔVIC	cactcctccagcaggaagcaagccccgcccaac	gttgggcggggcttgcttcctgctggaggagtg
γB4-ΔVIC1	ctgcgacactcctccagcccgtattcctacaatctgtgtgct	agcacacagattgtaggaatacgggctggaggagtgtcgcag
γB4-ΔVIC2	aactacagtgaggggactttgcaagatctacaatttgcagattca	tgaatctgcaaattgtagatcttgcaaagtcccctcactgtagtt
γB4-ΔVIC3	acatgtaatgatcagttgaaatcaggacaagccccgcccaacact	agtgttgggcggggcttgtcctgatttcaactgatcattacatgt
