# Supplementary material for: Structural insights into the in situ assembly of clustered protocadherin γB4
Source: Nat Commun. 2025 Feb 16;16:1682. doi: 10.1038/s41467-025-56948-x (PMC11830823; doi:10.1038/s41467-025-56948-x)
Supplement: Supplementary file 2 — Description of Additional Supplementary Files [file 41467_2025_56948_MOESM2_ESM.pdf]

Description of Additional Supplementary Files

File Name: Supplementary Movie 1
Description: A tomogram and 3D model of the cell adhesion interface by γB4-ΔIC
